# VAR2CSA-Mediated Host Defense Evasion of *Plasmodium falciparum* Infected Erythrocytes in Placental Malaria

**DOI:** 10.3389/fimmu.2020.624126

**Published:** 2021-02-09

**Authors:** Alice Tomlinson, Jean-Philippe Semblat, Benoît Gamain, Arnaud Chêne

**Affiliations:** ^1^ Université de Paris, Biologie Intégrée du Globule Rouge, UMR_S1134, BIGR, INSERM, Paris, France; ^2^ Institut National de la Transfusion Sanguine, Paris, France; ^3^ Laboratory of Excellence GR-Ex, Paris, France

**Keywords:** *Plasmodium falciparum*, placental malaria, VAR2CSA, PfEMP1, immune evasion, immuno-modulation, VAR2CSA polymorphism

## Abstract

Over 30 million women living in *P. falciparum* endemic areas are at risk of developing malaria during pregnancy every year. Placental malaria is characterized by massive accumulation of infected erythrocytes in the intervillous space of the placenta, accompanied by infiltration of immune cells, particularly monocytes. The consequent local inflammation and the obstruction of the maternofetal exchanges can lead to severe clinical outcomes for both mother and child. Even if protection against the disease can gradually be acquired following successive pregnancies, the malaria parasite has developed a large panel of evasion mechanisms to escape from host defense mechanisms and manipulate the immune system to its advantage. Infected erythrocytes isolated from placentas of women suffering from placental malaria present a unique phenotype and express the pregnancy-specific variant VAR2CSA of the *Plasmodium falciparum* Erythrocyte Membrane Protein (PfEMP1) family at their surface. The polymorphic VAR2CSA protein is able to mediate the interaction of infected erythrocytes with a variety of host cells including placental syncytiotrophoblasts and leukocytes but also with components of the immune system such as non-specific IgM. This review summarizes the described VAR2CSA-mediated host defense evasion mechanisms employed by the parasite during placental malaria to ensure its survival and persistence.

## Introduction

Nearly half the world’s population, implicating 90 countries, lives in areas at risk of malaria transmission. In 2019, an estimated 11 million pregnant women were infected by *Plasmodium* in sub-Saharan Africa, where *P. falciparum* is the most prevalent parasite species, accounting for 99.7% of estimated malaria cases (1). *P. falciparum* infection contracted during pregnancy can lead to placental malaria (PM), a condition that could cause very serious clinical outcomes for both mother and child, including maternal anemia (2, 3), hypertension (4, 5), stillbirth (6, 7) as well as low birth-weight infants, which affected over 800,000 children in 2019 (1).

**Figure 1:**
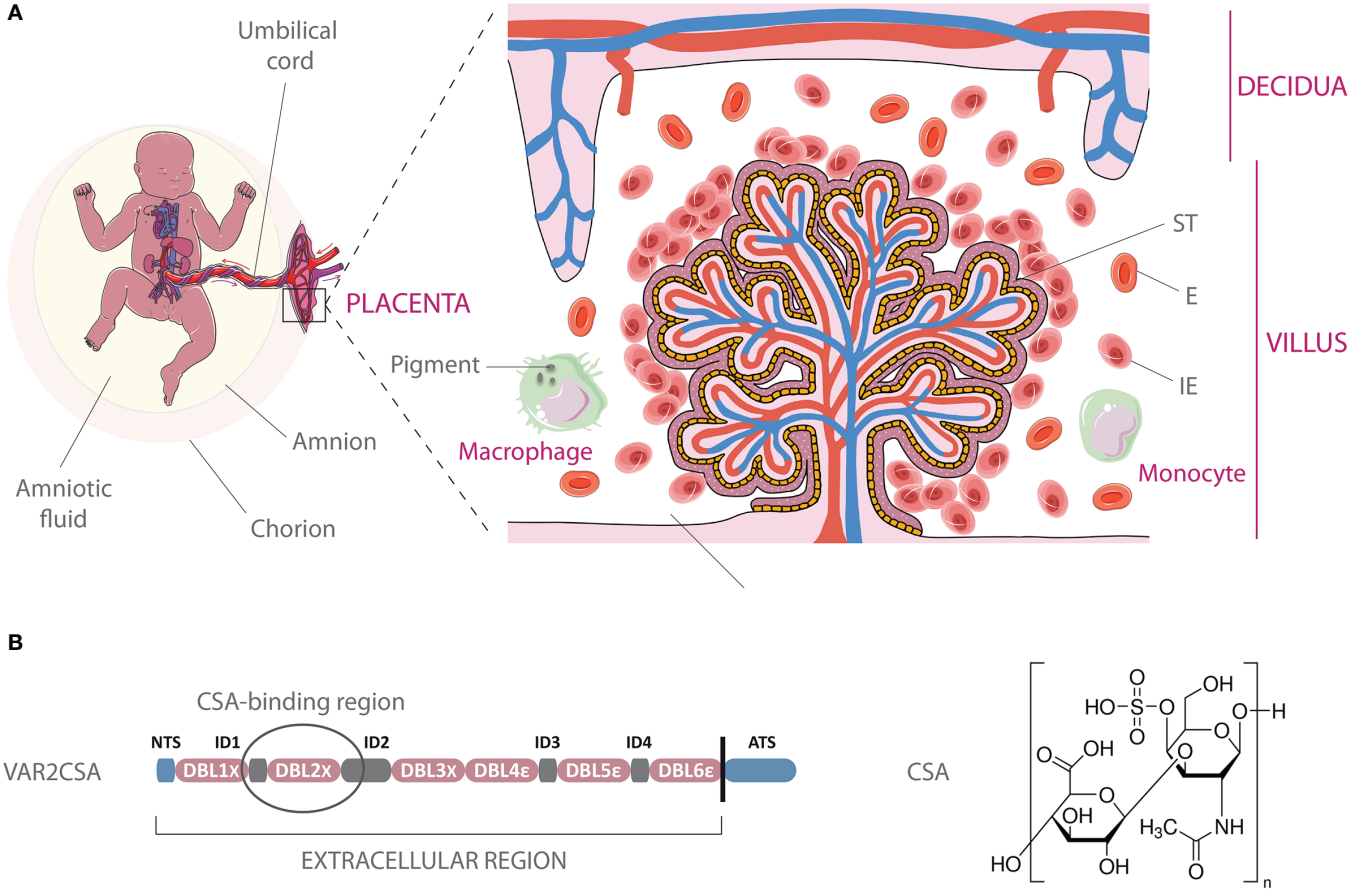
Infected erythrocyte sequestration within the intervillous space of the placenta. **(A)** Schematic representation of infected erythrocytes (IE) adhering to the syncytiotrophoblastic lining of the fetal villus, with increased presence of macrophages and monocytes in the maternal blood. Parasite pigments (hemozoin) remain visible in macrophages following IEs phagocytosis. The natural transfer of gases and nutrients between maternal blood in the intervillous space and fetal blood circulating in the villi is impaired by IEs sequestration. E, Erythrocyte; ST, syncytiotrophoblast. **(B)** Architecture of the VAR2CSA protein and chemical structure of chondroitin-4-sulfate A. The circled region within VAR2CSA (ID1-ID2a) represents the CSA-binding region. The art pieces used in this figure were modified from Servier Medical Art by Servier, licensed under a Creative Commons Attribution 3.0 Unported License (https://smart.servier.com/).

PM may result in significant morphological and immunological changes in the placenta. Focal syncytial necrosis, loss of syncytial microvilli, and proliferation of cytotrophoblastic cells are frequently observed as well as thickening of trophoblastic basement membranes together with the apparition of syncytial knots (8–10). Acute infection is also characterized by the substantial presence of infected erythrocytes (IEs) in the intervillous spaces of the placenta (**Figure 1A**).

Several transcriptomic and proteomic studies revealed that parasitized red blood cells isolated from *P. falciparum*-infected pregnant women display specific signatures, over-expressing a variety of different genes (11–13) and proteins (14–16) as compared to non-pregnancy-specific parasites. They also present a unique adhesive phenotype, interacting with chondroitin sulfate A (CSA), a low-sulfated glycosaminoglycan (GAG), which is the major host receptor involved in the adhesion of IEs to syncytiotrophoblastic cells (17–21) (**Figure 1B**). Chondroitin sulfate-proteoglycans (CSPGs) are present in the intervillous space of the placenta during the entire second and third trimesters and possibly during the latter part of the first trimester (22).

To date, the pregnancy-specific variant of the *Plasmodium falciparum* erythrocyte membrane protein 1 family (PfEMP1) VAR2CSA has been identified as the sole parasite-derived protein interacting with placental CSA (23–28).

This review focuses on the roles played by VAR2CSA in PM pathogenesis and introduces the latest information on its involvement in host defense evasion mechanisms ranging from cytoadhesion in the placenta, modulation of the placental microenvironment to escape of pregnancy-specific IEs from recognition by protective antibodies.

## VAR2CSA Structure and Chondroitin Sulfate A (CSA)-Binding

VAR2CSA is a large protein of 350 kDa, with an extracellular region of approximately 300 kDa, displayed at the surface of IEs on membrane protrusions called knobs (29). PfEMP1 clustering on knob structures is thought to maximize cytoadhesion under flow conditions but also to act as an immune evasion mechanism, impairing antibody accessibility to key residues involved in CSA-binding (30, 31). Quantitative studies report an estimate of 3 to 80 VAR2CSA molecules per knob (32, 33). Knob density at the IEs surface has been shown to be linked to the PfEMP1 variant expressed by the parasite (34) and IEs stained by the monoclonal antibody PAM1.4 revealed that erythrocytes infected by the FCR3 parasite strains displayed more VAR2CSA clusters at the cell surface than erythrocytes infected by NF54 (35). Even if further studies are needed to precisely determine how these differences in PfEMP1 presentation impact antibody recognition, these observations highlight that *P. falciparum* is capable of complex variations at both intra- and inter-strain levels.

The cysteine-rich extracellular region of VAR2CSA has a complex architecture and is composed of six Duffy-Binding Like domains (DBLs), which are interspaced by four inter-domain regions (IDs) (**Figure 1B**). High-resolution structures have been obtained for the individual domains DBL3x, DBL6ϵ (36–40) as well as for the multidomain DBL3x-DBL4ϵ (41), providing a first step towards the definition of inter-domain interfaces and of the overall structure of the extracellular part of VAR2CSA. Low-resolution structures of the full-length extracellular part of VAR2CSA, obtained by small-angle X-ray scattering or single particle electron microscopy, reveal a compact organization of the protein maintained by specific inter-domain interactions (42–44). Nevertheless, the relative locations of the DBL domains within the overall structure of VAR2CSA significantly differ from one study to another (43, 44). In the recent work from Bewley et al., the VAR2CSA ectodomain low resolution structure appears as a duck-like shape with a packing of three tandem domains (DBL1x/DBL2x, DBL3x/DBL4ϵ, and DBL5ϵ/DBL6ϵ), which would form two pores, each theoretically susceptible to accommodate a 10-12-mer CSA. molecule (44). This model suggests that the higher-order structural organization of VAR2CSA is most likely allowing the formation of one, or maybe two, CSA-binding site(s), which comprise(s) several domains. The current definition of the boundaries of the core binding region, established using truncated fragments of recombinant VAR2CSA, localizes the high affinity CSA-binding site within the N-terminal part of the protein (45) between the ID1-ID2a section (46) even-though the accessory implication of other domains such as DBL4ϵ cannot be excluded (44). Additional VAR2CSA structural data at high resolution, ideally in complex with CSA, is still required to determine the precise determinants of CSA-binding, which might also include post-translational modifications (47).

## VAR2CSA-Mediated Infected Erythrocytes Cytoadhesion in The Placenta and Evasion from Splenic Filtration

As parasites develop from ring stage to schizont stage within erythrocytes, the biomechanical properties of the host cells are subjected to considerable modifications, leading to decreased cellular deformability and loss of membrane elasticity [Reviewed in (48)]. Cytoadhesion of mature pregnancy-specific IEs to syncytiotrophoblasts leads to their sequestration in the intervillous spaces of the placenta. By sequestering in the placenta, biomechanically altered IEs avoid splenic retention at the level of the reticular mesh of the red pulp or during the challenging passage through the inter-endothelial slits of the organ (49–52). *P. falciparum* has therefore developed an efficient host defense evasion mechanism, which relies on a tight interaction between IEs and the syncytiotrophoblastic lining delimiting the intervillous spaces of the maternal portion of the placenta. As CSPGs are also present within the micro-vascular system, notably in the lungs and brain (53), the reason for exclusive placental sequestration of VAR2CSA-expressing IEs remains unclear. A body of work elucidated some comprehensive elements by demonstrating that the interaction of VAR2CSA with CSA is highly correlated with the degree of C-4 sulfation and the length of the CS chain (54–56), which may vary in different tissues. CSA density and wall shear stress also appear as two components influencing the IEs binding to CSA (57). CSA density on syncytiotrophoblasts and forces acting upon placental tissues could thus determine the selective cytoadhesion of IEs in the organ. If placental sequestration of IEs represents an effective immune evasion mechanism employed by *P. falciparum* to avoid its clearance by the spleen, this is not without harmful consequences for the women and the fetus. Sequestration is thought to be one of the prime mediators of biological alterations leading to placental insufficiency and subsequently to fetal growth restriction and poor birth outcomes [Reviewed in (58, 59)].

## VAR2CSA-Mediated Modulation of The Placental Microenvironment

The placenta is a tightly controlled pro-inflammatory and anti-inflammatory environment, depending upon the stage of gestation. In healthy pregnancies, a pro-inflammatory milieu is required for fetal implantation, notably by promoting trophoblast invasion. A shift toward a type 2 cytokine/chemokine milieu gradually occurs during gestation favoring pregnancy maintenance and rapid fetal growth and development [reviewed in (60)]. *P. falciparum* infection during pregnancy can affect the placental environment, notably promoting inflammatory responses (61–65), some of which are associated with fetal growth retardation, low birth-weight babies, and in more extreme cases, poor pregnancy outcomes, such as preterm delivery and pregnancy loss (66–71). *P. falciparum* is thus able to upset the fine equilibrium between pro-inflammatory and anti-inflammatory responses, deregulating the immune system, with detrimental consequences for the human host.

### Syncytiotrophoblast Activation

The syncytiotrophoblasts covering the placental villi are terminally differentiated cells, which result from the syncytialization of underlying villous cytotrophoblasts. They exhibit high metabolic activity and are involved in many physiological processes such as the active transport of molecules, the diffusion of gases, and the synthesis and secretion of large amounts of hormones, including steroids [Reviewed in (72)]. Experiments performed using primary placental cells, as well as the widely used choriocarcinoma cell line BeWo, revealed that VAR2CSA-dependent binding of IEs to syncytiotrophoblasts induces a broad range of cellular responses, notably activating MAPK pathways (73, 74). Activation of syncytiotrophoblasts leads to the secretion of pro-inflammatory cytokines/chemokines such as macrophage inflammatory protein (MIP), the neutrophil chemotactic factor interleukin (IL) 8 and IL-6 (74, 75), but also to the production of soluble ICAM-1 (75), which may act as a protection mechanism to regulate the inflammatory response (76). The interaction of syncytiotrophoblasts with VAR2CSA-expressing IEs might therefore participate in the immunological shaping of the local environment, establishing a complex network of factors which could promote the migration of immune cells to the intervillous space (74), as well as the *in situ* modulation of their activity.

### Macrophage and Monocyte Immunomodulation

Sections taken from healthy placenta at different time-points throughout normal pregnancy showed that nearly half of the decidual cells are of bone marrow origin, comprising 18–20% macrophages (77, 78). Polarization of decidual macrophages varies with gestational age, shifting from an M1 polarization during fetal implantation, towards a mixed M1/M2 profile which remains until mid-pregnancy (79). After the placental development is completed, decidual macrophages are predominantly of the M2 phenotype, contributing to a tolerant immune environment and to fetal immunoprotection (80, 81).

PM is characterized by a significant increase in the number of monocytes and macrophages in the intervillous space (8–10, 82, 83), which is notably associated with elevated expression of the β chemokines IL-8 and MIP-1 (84). *In vitro* co-incubation experiments, performed in absence of human plasma/serum, i.e. in absence of opsonic antibodies, showed that VAR2CSA-expressing IEs are able to modulate specific transcription factor activation in RAW-macrophages, as compared to erythrocytes infected with genetically modified parasites presenting a deficiency in the export of PfEMP1 at the cell surface (PfEMP1-null) (85). The decreased activation of NF-κB-, CREB-, and GAS/ISRE-binding factors is accompanied by reduced production of TNF and IL-10. Similar experiments using human primary monocytes also revealed that VAR2CSA-expressing IEs are able to alter the production profiles of other cytokines/chemokines, limiting the release of IL-1β, IL-6, IL-10, MCP-1, MIP-1α, and MIP-1β, as compared to cells infected withfimmu.2020.624126 PfEMP1-null parasites (85). Although the precise nature of the monocyte receptor(s) involved still remains to be elucidated, these observations highlight how *P. falciparum* could exploit the host cellular pathways to modulate the immune response.

Interestingly, a study performed in an area of low prevalence of malaria, revealed gravidity-dependent differences in the capacity of peripheral blood mononuclear cells (PBMCs) to produce cytokines and chemokines in response to pregnancy-specific IEs (86). Despite no differences in opsonic antibody levels, cellular immune responses differed between women in their second to fourth pregnancy (G2-4) and grand multigravida (G5-G7). Indeed, more IL-10, IL-1β, IL-6, tumor necrosis factor (TNF) but less CXCL-8, CCL-8, IFNγ, and CXCL-10 were detected in G2-4 compared to G5-7, highlighting the modulation of immune cell function occurring during PM (86).

## VAR2CSA Binding to Non-Specific IGM and Diversion of The Immune Response

PM induces VAR2CSA-specific immunoglobulin Gs (IgGs) belonging to the IgG1 subclass, and to a lower extent the IgG3 subclass (87, 88), both highly potent at interacting with Fcγ receptors present at the surface of phagocytic cells. Concordantly, women living in areas where malaria is endemic naturally acquire specific antibodies that promote the phagocytosis of VAR2CSA-expressing IEs (89–91), thus participating in parasite clearance. Binding of non-specific IgM on the surface of IEs was first demonstrated on rosetting parasites (92–94) and subsequently on VAR2CSA-expressing red blood cells (95). Following these observations, the function of IgM binding to VAR2CSA has been uncertain for several years. In 2011, a study performed by Barfod et al. showed that non-specific IgM binding participates in the masking of protective epitopes on VAR2CSA, leading to IE evasion of macrophage-mediated opsonic phagocytosis (96). The same study revealed that non-specific IgM binding to VAR2CSA-expressing IEs did not interfere with their capacity to adhere to CSA and did not increase their susceptibility to undergo complement-mediated lysis (96). The extensive analysis of non-specific IgM binding to large panels of PfEMP1 members demonstrated that IgM binding is a common functional phenotype found in multiple PfEMP1 variants across various parasite strains, thus providing a better understanding of the underlying molecular mechanisms (97–99). Although the CSA-binding site of VAR2CSA resides within the N-terminal region of the protein (100, 101), the IgM interacting residues appear to be mainly located within the C-terminal section, at the level of the DBL5ϵ or DBL6ϵ domains in VAR2CSA variants carried by the 3D7 and FCR3/IT parasite strains, respectively (102, 103) as well as in DBLϵ and DBLζ domains near the C-terminus of other PfEMP1 variants (98, 99, 104, 105).

The PfEMP1 binding sites on IgM have been located within the μ region of the fragment crystallizable (Fcμ) of polymeric immunoglobulins (97), and more precisely in the Cμ4 domain for the DBL4β domain of PfEMP1-VAR1 of the TM284 strain (106). These observations, together with the additional definition of the architecture of the IgM/PfEMP1 complex (107), provide critical molecular elements which could explain how PfEMP1s interfere with the binding of the complement component C1q to the adjacent Cμ3 domain, thus inhibiting complement-mediated lysis. Furthermore, these findings demonstrate how IgMs participate in PfEMP1 clustering on the cell surface, strengthening the interactions with host receptors (107–109). PfEMP1 binding to IgM has also been proposed as a non-exclusive molecular mechanism involved in the triggering of polyclonal B cell activation, a hall mark of malaria (110, 111). This activation would lead to hyper-gamma-immunoglobulinemia and the subsequent diversion of the specific humoral immune response towards antigens relevant for protection.

## VAR2CSA Polymorphism

All the *P. falciparum* genomes sequenced to date reveal the presence of one or more *var2csa* gene copies (112–114). VAR2CSA is a highly polymorphic multidomain protein, usually consisting of six DBL domains; the first three DBL domains belong to the DBLx subtype and the three others to the DBLϵ subtype. The protein also contains a CIDR_PAM_ domain (also referred to as ID2) between the DBL2x and DBL3x domains. A recent study has identified atypical extended or truncated VAR2CSA structures (115). Extended structures include one or two additional DBLε domains downstream of the conventional DBL1x-6ε domain structure (115). Within the conventional six DBL domain structure, DBL4ε is the most conserved DBL domain while DBL6ε is the most polymorphic DBL domain (112). *Var2csa* is present in all genomes of known *Laverania* sub-genus members (116). One of the closest *P. falciparum* relatives, the chimpanzee parasite *Plasmodium reichenowi*, possesses a *var2csa*-like gene which is annotated as a pseudogene and encodes a functional truncated protein (NTS-DBL1x-ID1-DBL2x-truncated ID2) (117).

Global sequence diversity and analysis of *var2csa* have been reported in different studies (118–121) and more recently for 1,249 sequences spanning 7 Kb of *var2csa* (NTS-DBL5ε) from various strains and field isolates (122). Although it was previously shown that the DBL6ε domain is the most polymorphic domain (112), this latest study, which does not include DBL6ε, demonstrates that the nucleotide diversity is higher towards the N-terminus of the protein and that the diversity is generally higher in African parasite populations than in South East Asian populations. While the DBL2x domain has the lowest nucleotide diversity (122), it possesses the highest density of insertions and deletions, with sequence length across samples ranging from 430 to 550 amino acids (122). In a population structure analysis performed on *var2csa* sequences from Benin and Malawi, five different clades of ID1-DBL2x (encoding for the CSA-binding region) were identified and the authors found an association between the 3D7-like clade and low birth-weight (120). Only four clades were identified, including a 3D7-like clade (clade 1) and an FCR3-like clade (clade 2) (120). Indeed, two of the previously identified clades could not be separated using this much larger dataset. Clades 1, 2, and 4 were present across all the *P. falciparum* malaria endemic areas and clade 1, which is associated with low birth-weight, is highly represented in the West African populations (41.7%), followed by East Africa (27.5%), South East Asia (23.5%), and South America (21.1%). However, clade 3 is exclusively found in African parasite populations but appears to represent less than 1% of the *var2csa* sequences.

A recent study, which used plasma obtained from Tanzanian and Malian women at the time of delivery, simultaneously examined the capability of antibodies to recognize native VAR2CSA expressed by either NF54 or FCR3, to inhibit the binding of IEs to CSA and to promote phagocytosis by THP1 cells. Plasma from Malian women reacted more strongly with VAR2CSA-expressing erythrocytes infected by the FCR3 parasite strain whereas Tanzanian plasma preferentially reacted with erythrocytes infected by NF54 (35). Analysis of antibody functionality showed that the balance between binding inhibition capability and opsonizing activity could be biased depending on the expressed VAR2CSA variant and on the geographical location (35), suggesting that epitopes involved in each functional process may differ among parasite strains and that parasite transmission in a given place could therefore shape antibody profiles. In addition, the multiplicity of *var2csa* genes within the parasite genome may also confer a greater capacity for antigenic variation and evasion of variant-specific immune responses (114).

## Concluding Remarks

**Figure 2:**
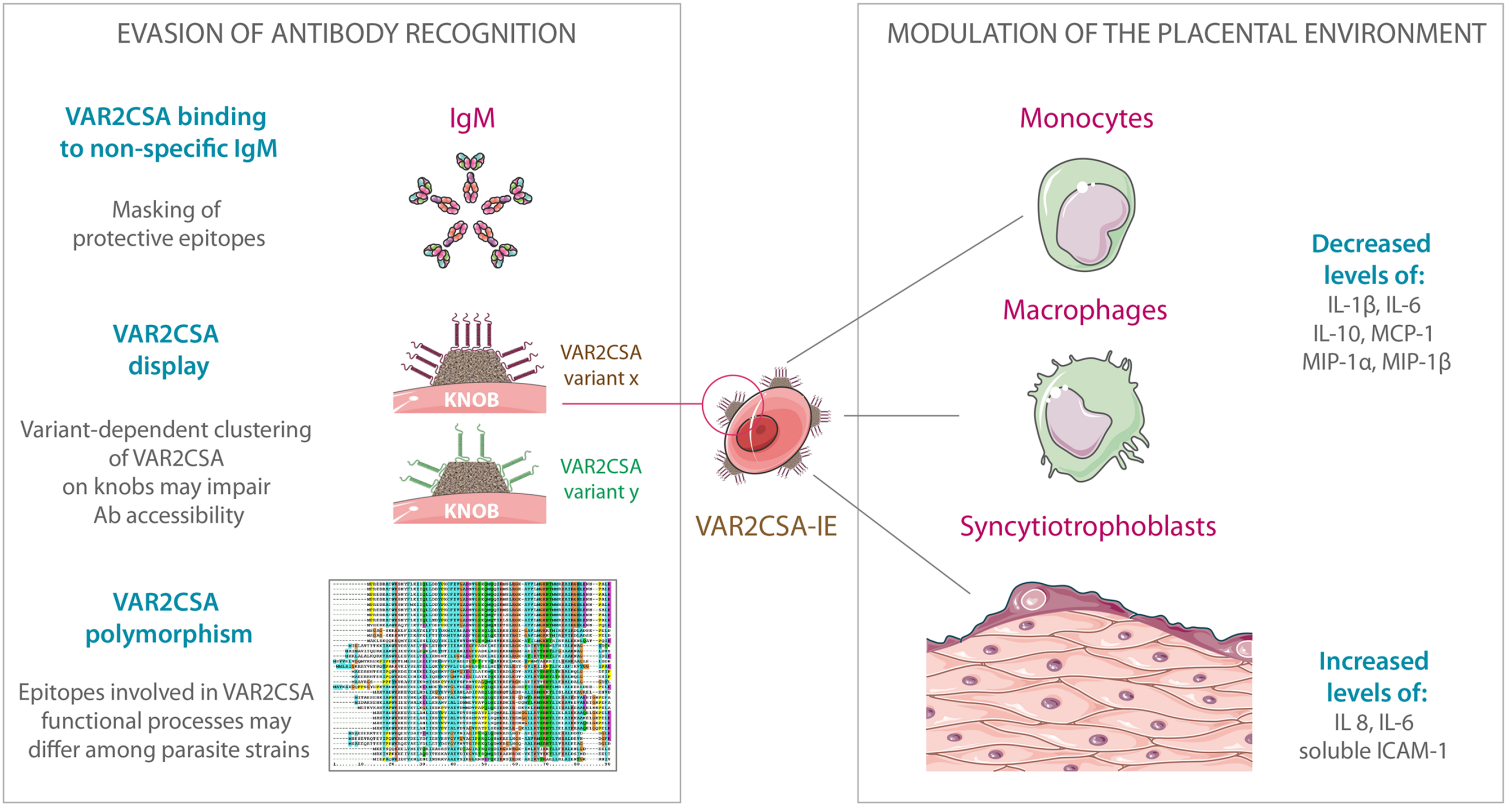
Evasion of antibody recognition and modulation of the placental environment by VAR2CSA-expressing infected erythrocytes. IgM binding to VAR2CSA could mask protein epitopes recognized by anti-VAR2CSA IgGs and consequently alter opsonic phagocytosis of IEs. PfEMP1 clustering on knob structures may act as an immune evasion mechanism, impairing antibody accessibility to key residues involved in CSA-binding. Due to extensive polymorphism, epitopes involved in each VAR2CSA functional process may differ among parasite strains. Furthermore, multiplicity of *var2csa* genes within the parasite genome may also confer a greater capacity for antigenic variation and evasion of variant-specific immune responses. The presence of VAR2CSA on the IEs surface could lead to decreased production of IL-1β, IL-6, IL-10, MCP-1, MIP-1α, and MIP-1β by monocytes and macrophages. VAR2CSA-dependent binding of IEs to syncytiotrophoblasts is able to activate MAPK pathways and lead to increased secretion of IL-8, IL-6, and soluble ICAM-1. The art pieces used in this figure were modified from Servier Medical Art by Servier, licensed under a Creative Commons Attribution 3.0 Unported License (https://smart.servier.com/). The illustration of the protein sequence alignment is licensed under a Creative Commons Attribution, CC BY-SA 3.0 (https://creativecommons.org/licenses/by-sa/3.0/).

*P. falciparum* infection contracted during pregnancy elicits a broad range of immune responses, combining components of both the innate and the adaptive immunity, orchestrated by a complex network of pro- and anti-inflammatory cytokines (**Figure 2**). *P. falciparum* has developed the ability to manipulate the immune system to its advantage to ensure its survival and persistence within the human host.

Although the parasite is able to escape host defense processes and manipulate the induced immune response using a variety of mechanisms described herein, women living in malaria endemic areas can gradually acquire protective clinical immunity against PM, depending on the intensity of parasite transmission (123). In moderate malaria transmission. PM adverse clinical outcomes can be seen in women of all parity status (124), whereas protection appears to develop in a more marked parity-dependent manner in high transmission settings (125). Importantly, PM protection has been linked to the presence of antibodies targeting PM-specific variant surface antigens (126) and more specifically VAR2CSA (127–129). These observations led to the belief that a VAR2CSA-based vaccine against PM could potentially be achieved. However, the high degree of sequence diversity within VAR2CSA represents a major hurdle for vaccine design.

Following extensive preclinical evaluation, two recombinant vaccine candidates PRIMVAC and PAMVAC, comprising the CSA-binding region of VAR2CSA from the 3D7 (clade 1) and FCR3 (clade 2) strains respectively, have been assessed in Phase I clinical trials in Europe and Africa (ClinicalTrials.gov identifiers NCT02658253 and NCT02647489, respectively) (130–134). The identification of immunological correlates of protection against PM being complex, there is to date no clear surrogate allowing an easy evaluation of the protective effects of vaccines in early clinical trials (135). Exploratory analyses performed for the PRIMVAC and PAMVAC trials nevertheless revealed that vaccine-induced antibodies had a limited capability to cross-react with VAR2CSA originating from heterologous parasite strains (133, 134), highlighting the difficulty to compose with the high degree of polymorphisms of the protein when designing vaccines. Alternative vaccine approaches, using VAR2CSA in combination with other *P. falciparum* antigens, such as the circumsporozoite protein (CSP) (136), or virus-like particles (VLPs) to display VAR2CSA-derived antigens are also currently under investigation (137–139).

Improving our understanding on how *P. falciparum* escapes host defenses, modulates the immune system and on how natural immunity develops during PM despite VAR2CSA polymorphisms is therefore crucial to design efficient and effective immuno-therapeutic approaches but also to appropriately evaluate them.

## Author Contributions

AT, J-PS, BG, and AC wrote the manuscript. All authors contributed to the article and approved the submitted version.

## Funding

This work was supported by a grant from the ANR-18-IDEX-0001, IdEx Université de Paris attributed to AT and BG.

## Conflict of Interest

The authors declare that the research was conducted in the absence of any commercial or financial relationships that could be construed as a potential conflict of interest
